# Single-versus double-layer uterine closure at the time of cesarean delivery and risk of uterine scar niche: a systematic review and meta-analysis of randomized trials

**DOI:** 10.1007/s00404-025-08151-y

**Published:** 2025-08-20

**Authors:** Mattia Dominoni, Marco Torella, Rossella Molitierno, Mario Fordellone, Gabriele Saccone, Dario Colacurci, Antonio Simone Laganà, Martina Rita Pano, Barbara Gardella, Marco La Verde

**Affiliations:** 1 Department of Woman, Child and General and Specialized Surgery, Obstetrics and Gynaecology Unit, University of Campania “Luigi Vanvitelli”, Naples, Italy; 2 Medical Statistics Unit, University of Campania Luigi Vanvitelli, Naples, Italy; 3 Department of Neuroscience, Reproductive Sciences and Dentistry, School of Medicine, University of Naples Federico II, Naples, Italy; 4 Department of Public Health, School of Medicine, University of Naples Federico II, Naples, Italy; 5 Unit of Obstetrics and Gynecology, Department of Health Promotion, Mother and Child Care, Internal Medicine and Medical Specialties (PROMISE), “Paolo Giaccone” Hospital, University of Palermo, 90127 Palermo, Italy; 6 Department of Clinical, Surgical, Diagnostic and Paediatric Sciences, University of Pavia, Corso Strada Nuova, 65, 27100 Pavia, Italy; 7 Department of Obstetrics and Gynecology, Fondazione IRCCS Policlinico San Matteo, Viale Camillo Golgi 19, 27100 Pavia, Italy

**Keywords:** Cesarean, Cesarean section, Ultrasound, Uterine closure, Uterine scar defect, Scar defects, Isthmocele, Niche, Double-layer, Single-layer

## Abstract

**Objective:**

This systematic review and meta-analysis compared single- versus double-layer uterine closure at the time of cesarean delivery.

**Data sources:**

MEDLINE, EMBASE, Scopus, ClinicalTrials.gov, and Cochrane Central Register of Controlled Trials were searched from inception until May 2024.

**Study eligibility criteria:**

We included only randomized controlled trials (RTSs) that compared single-layer versus double-layer uterine closure at the time of cesarean delivery.

**Study appraisal and synthesis methods:**

Outcomes were analyzed using a random-effects model. Results are expressed as risk differences. The assessment of the risk of bias was performed by using the Risk of Bias 2 tool. The primary outcome was the incidence of scar defects (i.e., niche) at 6 months after delivery. The secondary outcomes were incidence of scar defects at 6 weeks and 3 months.

**Results:**

A total of 18 studies were identified by the systematic review; 11 RCTs involving 6,058 participants were included in the meta-analysis. There is no statistical difference between single-layer and double-layer uterine closure of cesarean delivery incision regarding the incidence of uterine scar defect at six weeks. Single-layer closure showed a significantly lower incidence of niche after three months (RD = − 0.02 (− 0.06, 0.02); *I*^2^ = 81%, *p* < 0.01), and six months (RD = − 0.11, CI − 0.15, − 0.07, I^2^ = 91%, *p* < 0.01).

**Conclusions:**

Single-layer uterine closure at the time of cesarean delivery resulted in a lower uterine scar defects after three and six months compared to double-layer uterine closure.

**Systematic review registration:**

PROSPERO, Unique identifier: CRD42024552495.

**Supplementary Information:**

The online version contains supplementary material available at 10.1007/s00404-025-08151-y.

## Introduction

The global rise of Cesarean delivery (CD) rates has increased the interest in the short- and long-term related complications [1]. Postpartum hemorrhage, surgical site infection, and urinary tract injury represent the short-term complications [2]. The long-term complications involve conditions such as uterine scar defects, abnormal uterine bleeding, preterm birth, and an increased risk of uterine rupture in following pregnancies [3, 4]. Several studies confirm that, with an increased trend of CDs, both incidences of placenta accreta spectrum (PAS) and uterine scar-related issues of abnormal bleeding and reproductive complications increased [5, 6]. The incidence of uterine scar defects (i.e., uterine niche) after previous CD is highly variable in the literature, ranging approximately between 24 and 70% [7]. A niche or scar defect is defined as a defect of the site of a Cesarean section scar detected through an imaging technique, either with a transvaginal ultrasound (TVS) or sonohysterography, appearing as an anechoic triangular area within a residual myometrial thickness (RMT) [8]. TVS has emerged as an essential technique in the diagnosis of a uterine niche, especially among nonpregnant women who have previously performed a CD [9]. TVS can evaluate the diameters of the niche in terms of depth, length, and RMT [10]. According to Feldman et al., TVS detected a niche presence in 25.6 % of the cases [11]. The European Niche Taskforce has formulated uniform diagnostic criteria and a uniform measurement method for the sonographic assessment of uterine niche in non-pregnant women using a modified Delphi procedure [12]. Fifteen experts participated in this research and, by consensus, reached specific statements regarding the definition of a niche and its measurement. According to this study, a niche was defined as a defect in the site of a cesarean section scar identified by the depth of indentation at or greater than 2 mm [12]. The key measurements were the length of the niche in the sagittal and depth, RMT in the sagittal plane, and the transverse plane [12]. The niches relate to several complications, including abnormal uterine bleeding and abnormal spotting, and have consequences for future pregnancies [13]. The origin of the niche is multifactorial, probably a combination of technical, anatomic, and patient factors [14, 15]. The surgical technique used for uterine closure during CD influences uterine scar healing and RMT [15]. There are different techniques to close the uterus after CD incision: single- or double-layer closures with or without locking and either passing through or avoiding the decidua [16]. The evidence base relating to these techniques is heterogeneous and there is still no consensus about the optimal method [17]. A meta-analysis by Di Spiezio Sardo et al. in 2017 did not find a difference in the incidence of scar defects after the single-layer uterine closure technique versus the double-layer. They showed only a significantly thinner RMT on ultrasound with single-layer uterine closure [18]. A subsequent meta-analysis addressed the single-layer vs double-layer uterine closure technique after cesarean section and demonstrated that double-layer closure has been associated with greater MST and a lower incidence of dysmenorrhea [19]. Single-layer closure showed a shorter operation time [19]. Both techniques did not differ significantly in uterine dehiscence, healing ratio, maternal infections, and duration of hospital stay [19].

## Objective

This meta-analysis of randomized trials (RCTs) aimed to compare the risk of uterine scar defect after single-layer versus double-layer uterine closure at the time of cesarean delivery.

## Methods

This systematic review followed the PRISMA Checklist [20] and Cochrane Handbook for Systematic Reviews (version 6.2) [21]. The protocol was preregistered on PROSPERO [CRD42024552495]. Since this review concerned only published studies, ethical approval was not required. All data generated or analyzed during this study was included in this article and its supplementary information files.

### Eligibility criteria, information sources, search strategy

Randomized controlled trials were considered eligible if they met the following criteria: (I) description of patients > 18 years who underwent cesarean section, applying the single or double layer suture for uterine breach reparation; (II) the effect of single-vs double-layer uterine closure at the time of low transverse cesarean section on the risk of uterine scar defect evaluated on ultrasound, hysterography or sonohysterography; (III) implication of the two approaches for uterine defect closure on the risk of uterine dehiscence in the following pregnancy; (IV) depth of niche, adjacent myometrial thickness (AMT) and RMT related to the two approaches for uterine defect closure. The exclusion criteria were: (I) non-English articles; (II) not full-text available; (III) RCTs reporting results on single versus double-layer uterine techniques at follow-ups other than six weeks, three months, and six months; (IV); (V) Trials not describing outcomes of interest. A literature search from database inception to May 2024 was performed through the electronic databases MEDLINE, EMBASE, Scopus, ClinicalTrials.gov and Cochrane Central Register of Controlled Trials, while seeking a combination of the following keywords: ‘Cesarean’, ‘delivery’, ‘scar’, ‘defect’, ‘niche’, ‘isthmocele’, ‘pouch’, ‘dehiscence’; ‘uterine closure’, ‘closure’, ‘layer’ (Online Appendix 1).

### Study selection

**Fig. 1:**
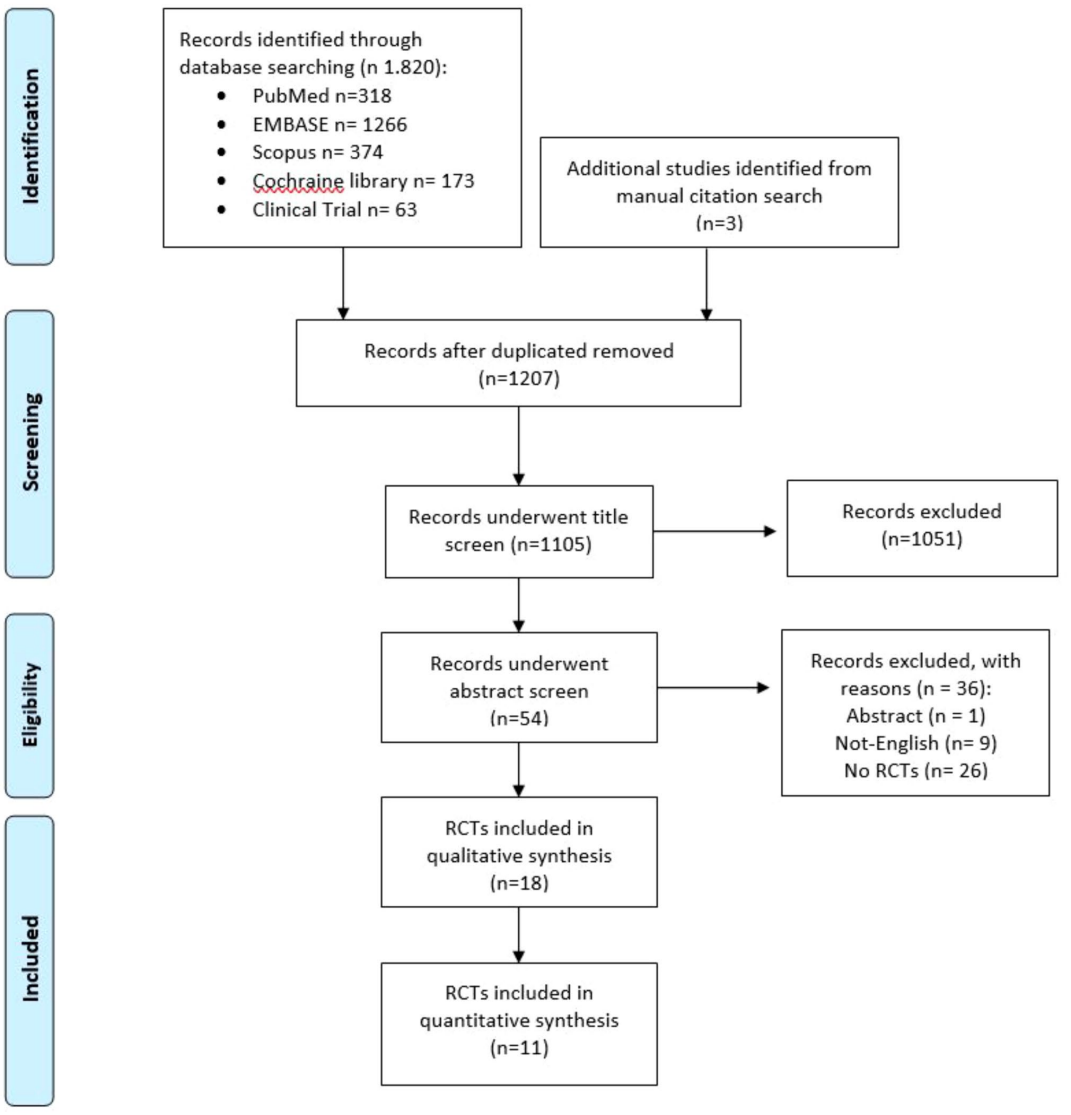
PRISMA flow chart of included studies

We considered RCTs published online until First of May 2024, analyzing the different suture techniques, single layer or double layer, for the low transverse Cesarean section reparation. The included studies evaluated the risk of uterine scar defect development after CD through ultrasound, hysterography, or sonohysterography. Study selection was conducted by two independent authors (D.C., L.M.). Both reviewers were blinded to each other’s evaluation, and after unblinding, disagreement was resolved through discussion or arbitration by senior author (M.L.V.). Each study was selected independently: first by title, second by abstract, and finally by full-text article (Fig. 1). All eligible RCTs were assessed using the modified checklist concerning the trial integrity assessment proposed by Weibel et al. [22]. RCTs with potential integrity concerns were identified and classified as ‘studies awaiting classification’, and not included for risk of bias assessment, data extraction, or analysis.

### Data extraction

Data were independently extracted by two authors (DC and LM). Any discrepancies were resolved through discussion or conferring with a third author (MLV). The characteristics to be extracted using the data extraction form included study characteristics (authors, year, country, and sample size); intervention characteristics (CD type, type of uterine closure); study outcomes and CD scar evaluation method; secondary outcomes (depth of niche, RMT, and AMT at ultrasounds and uterine dehiscence in next pregnancy). Authors of included studies were contacted for additional information about the primary outcomes analyzed.

### Assessment of risk of bias

**Fig. 2:**
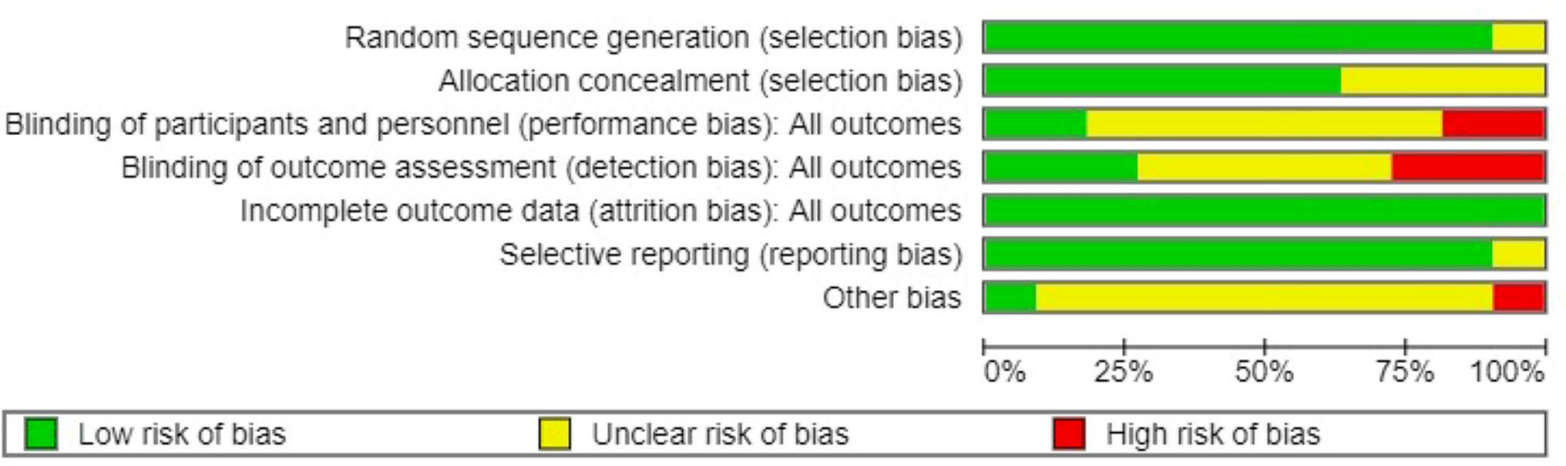
Summary of the Risk of bias according to Cochrane Handbook in randomized controlled trials included in meta-analysis

**Fig. 3:**
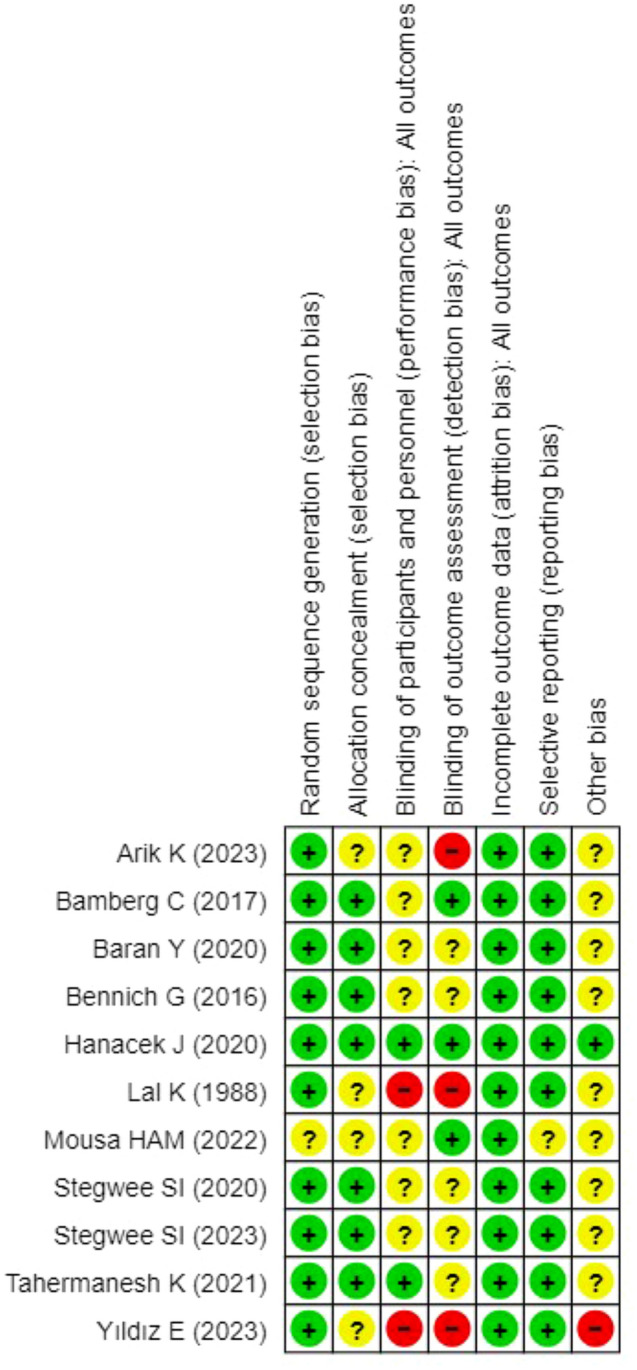
Figure 2 Risk of bias according to Cochrane Handbook in randomized controlled trials included in meta-analysis

The risk of bias (ROB) of the included study was assessed, referring to the criteria described in Cochrane Handbook for Systematic Reviews of Interventions [21]; seven domains were assessed: (1) generation of random sequence; (2) allocation concealment; (3) blinding of participants and personnel; (4) blinding of outcome assessment; (5) incomplete outcome data; (6) selective reporting; and (7) other bias. The judgements of review authors regarding the ROB were categorized as ‘ low risk’, ’ ‘ high risk’ or ‘ unclear risk'.’ Assessment of ROB was performed by two authors (D.C. and L.M.) and was supervised by two senior authors (M.D. and M.L.V.) Figure 2 and Figure 3 report the graphical classification of ROB for each included study and the ROB summary*.* We considered follow-up to all outcomes, with a minimum of six weeks and up to 6 months, excluding uterine dehiscence in the subsequent pregnancy.

### Data synthesis

This meta-analysis estimates the risk differences in several RCTs performing three separate meta-analyses at 6 weeks, 3 months, and 6 months post-treatment. Data are synthesized using both fixed-effect model and the random-effects model. In the fixed-effect model, the effect size is assumed to be identical in all RCTs, focusing on within-study precision and disregarding between-study variability. The random-effects model takes heterogeneity within and between RCTs into account, thus generalizing inferences about the true effect size. The I^2^ statistic is a measure of heterogeneity between RCTs and describes the percentage of variation among the effect estimates due not to random chance but because of between-study differences. τ^2^ statistic estimates between-study variance, and a p-value is calculated for testing the null hypothesis of homogeneity. High I^2^ and τ^2^ values, coupled with a significant *p*-value (*p* < 0.05), suggest substantial heterogeneity, indicating that the effect sizes differ considerably across the included studies. Secondary outcomes analysis in 6 weeks, 3 months, and 6 months post-CD was not possible due to the availability of few studies. The data management, initial formatting, and table generation was performed with R Studio Statistical software, version 4.1.3, and the statistical analysis was performed using STATA 16 software (Stata Statistical Software: Release 16. StataCorp LLC, College Station, TX, USA).

## Results

### Study selection and study characteristics

Out of 1823 studies identified, 18 studies were selected for systematic review of literature [23–40], while 11 RCTs were included in meta-analysis [23–34], as reported in Fig. 1. Overall, the analysis of primary outcomes, scar defect, considered 11 trials [23–34], 6058 participants, 3136 subjects in single-layer group and 2922 subjects in double-layer group, respectively. The secondary outcomes were reported in 13 RCTs [23–25, 28–31, 34, 35, 37–40], which included a total of 7083 subjects. Five trials for depth of niche [23, 25, 28–30], three trials for the incidence of uterine dehiscence in the subsequent pregnancy [36, 37, 39], six trials for the AMT [24, 25, 28, 30, 35, 40], and 12 trials for RMT [23–25, 28–31, 34, 35, 37, 38, 40]. The characteristics of the included studies are listed in Table 1. Cesarean-scar evaluation was performed by transvaginal ultrasound in 13 RCTs [23–31, 33–35, 40], by transabdominal ultrasound in 2 RCTs [37, 38], two RCTs adopted saline infusion sonography (SIS) [24, 35], one RCT adopted hysterography [32] and in two studies, it was not reported (Table 1) [36, 39]. Fifteen trials included a primary cesarean section in singletons [24–30, 32–36, 38–40]. Four studies also included secondary cesarean section [32, 36, 37, 39] (Table 1). Regarding single-layer uterine closure, 12 trials did not report the inclusion/exclusion of the decidual layer [23, 24, 33, 39]. Due to the limited number of RCTs stratified by follow-up time, quantitative analysis for the secondary outcomes (depth of niche, the incidence of uterine dehiscence in the subsequent pregnancy, AMT and RMT) was not available.

**Table 1:** Characteristics of randomized controlled trials meeting eligibility criteria

				Type of uterine closure	Type of uterine closure		
Authors (year)	Country	Sample size (*n*)	Inclusion criteria	Single-layer	Double-layer	Outcomes	CD scar evaluation method
Lal (1988)	India	100	Primary or secondary CD	Interrupted excluding decidua	Continuous unlocked	CD scar defect	Hysterography 3 months post CD
Chapman (1997)	USA	145	Primary or secondary CD	Continuous locked including decidua	Continuous locked	Uterine dehiscence in next pregnancy	NA
Borowski (2007)	USA	46	Planned primary CD in singletons	NA	NA	CD scar defect	TVS 6 weeks post CD
Yasmin (2011)	Pakistan	90	Secondary CD in singletons	Continuous locked including decidua	Continuous locked or unlocked	RMT, uterine dehiscence in next pregnancy	TAS 6 weeks post CD
El-Gharib (2013)	Egypt	150	Primary CD in singletons	Continuous locked including decidua	Continuous locked	RMT	TAS 2 weeks post CD
CORONIS (2016)	Multicenter	3234	Primary or secondary CD	Any method	Any method	Uterine dehiscence in next pregnancy	NR
Bennich (2016)	Denmark	61	Planned primary CD	Continuous unlocked including decidua	Continuous unlocked	RMT, CD scar defect	TVS 5 months post CD
Roberge (2016)	Canada	73	Planned primary CD in singletons	Continuous locked including decidua	Continuous locked or unlocked	RMT, CD scar defect	TVS 6 months post CD
Bamberg C (2017)	Berlin, Germany	435	Planned CD or non emergent	Continuous unlocked and locked single-layer	Continuous unlocked double-layer	RMT, CD scar defect	TVS 6 weeks and 6 months post CD
Yılmaz Baran Ş (2020)	Turkey	225	Primary CD	locked single-layer	locked double-layer	RMT, CD scar defect, AMT	SIS 6 months post CD
Yıldız E (2023)	Turkey	111	Plannede Primary CD	Continuous unlocked single-layer	NA	RMT, CD scar defect, AMT	TVS 6 weeks post CD
Hanacek J (2020)	Prague	540	Primary CD	Continuous unlocked single-layer	Continuous unlocked double-layer	CD scar defect	TVS 6 weeks and 6 months post CD
Stegwee SI (2023)	Netherlands	1961	Primary CD	NA	NA	CD scar defect	TVS 6 months post CD
Tahermanesh K (2021)	Iran	72	Primary CD	NA	NA	RMT, CD scar defect, AMT	TVS 3 months post CD
Stegwee SI (2020)	Netherlands	2292	Primary CD	Continuous unlocked single-layer	Continuous unlocked double-layer	RMT, CD scar defect	TVS 3 months post CD
Hanan Abdelwahab Meselhy Mousa; (2022)	Egypt	74	Planned Primary CD	NA	NA	RMT, CD scar defect, AMT	TVS 3 months post CD
Mohamed Kandil et al.; (2023)	Egypt	126	Primary CD	NA	NA	RMT, CD scar defect, AMT	TVS/SIS 6 months post CD
Arık M (2023)	Turkey	139	Planned CD	Continuous locked single-layer	Continuous locked double-layer	RMT, CD scar defect	TVS 6 months post CD

*CD* cesarean delivery, *RMT* residual myometrial thickness, *AMT* adjacent myometrial thickness, *TVS* Transvaginal ultrasound, *TAS* transabdominal ultrasound, *SIS* sonohysterography

### Risk of bias of included studies

The risk of bias assessment of included trials is generally low, as shown in Fig. 2. All the studies had a low risk in random sequence generation, incomplete outcome data, and selective reporting. Adequate methods for allocation of concealment were reported.

### Synthesis of the results

#### Six weeks after cesarean section

**Fig. 4:**
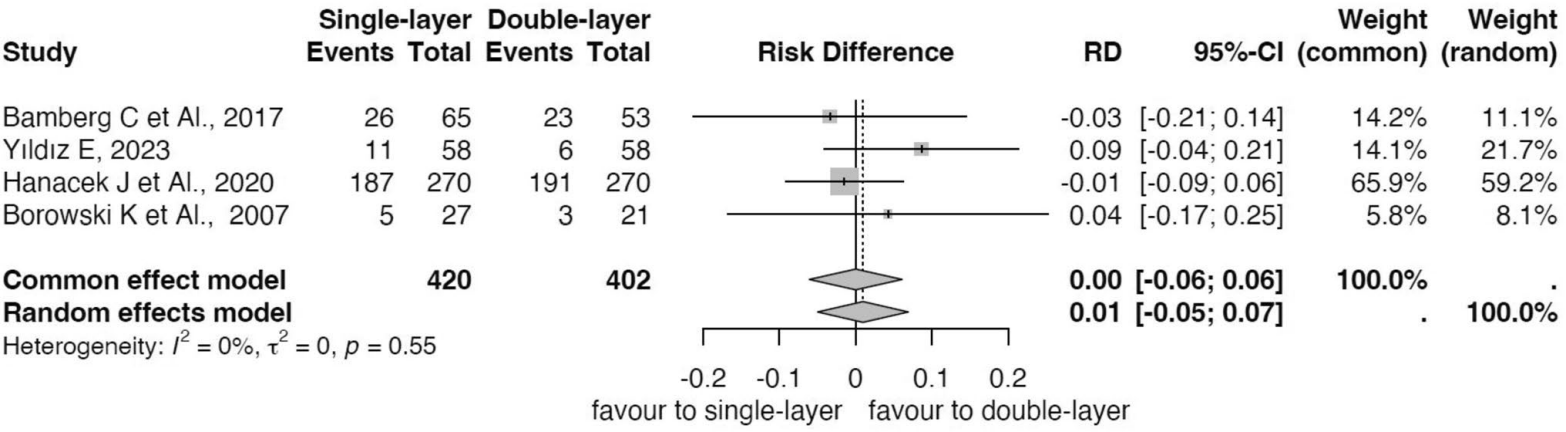
Forest plots showed niche incidence at six weeks post-cesarean section

Table 1 shows the characteristics of the studies included and Table 2 shows the secondary outcomes data. Single-layer uterine closure and double-layer closure had no statistical difference in the incidence of uterine scar defects six weeks after CD (Risk Difference (RD) = 0.00, CI − 0.06;0.06, Fig. 4) in four trials with 822 subjects (420 in single-layer group and 402 in double layer group). Six weeks after the cesarean section, the niches were reported in 229 subjects in the single-layer group and 223 subjects in the double-layer group, respectively. Heterogeneity was low I^2^ = 0% and *p* = 0.55 (Fig. 4). Two studies reported the niche depth [23, 25], four RMT values [23, 25, 37, 38] with a minimum value of 4.8 mm ± 0,8 mm and 17.08 mm ± 1.635 mm as maximum value of 17.08 mm ± 1.635 mm reported (Table 2). One study reported AMT in a value of 10.3 mm ± 1.3 mm for single-layer group and 11.7 ± 1.5 for the double-layer group [25] (Table 2).

**Table 2:** Secondary outcomes in randomized controlled trials comparing effect of single- vs double-layer uterine closure after six weeks of Cesarean delivery (CD): depth of niche, residual myometrial thickness (RMT) and adjacent myometrial thickness (AMT)

Outcomes 6wks	Trials (n.refs)	Participants (*n*)	Single-layer closure*	Double-layer closure*
Depth of niche (mm ± DS)	2 [23, 25]	546	3.0 + 1.4 4.2 ± 0.7	3.3 ± 1.3 4.5 ± 1.0
RMT at US (mm)	4 [23, 25, 37, 38]	786	5.7 ± 4.4 5.4 ± 0.6	7.2 ± 3.8 6 ± 0.5
AMT at US (mm) (mm ± DS)	1 [25]	111	10.3 ± 1.3	11.7 ± 1.3

*RMT* residual myometrial thickness, *AMT* adjacent myometrial thickness

#### Three months after cesarean section

**Fig. 5:**
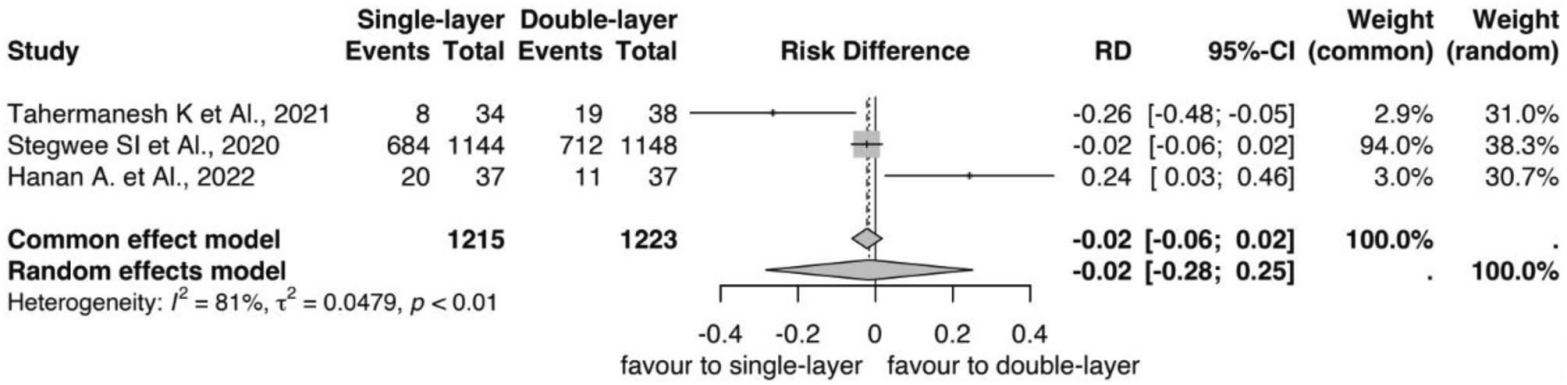
Forest plots showed niche incidence at three months post-cesarean section

Considering 3 months after CD, as reported by 3 trials [28–30] with 2438 women (1215 women in single layer arm, and 1223 in double layer arm, Fig. 5), women who received single-layer closure had a reduced risk of insurgence of niches (RD = − 0.02; CI − 0.06,0.02; *p* < 0.01 Fig. 5). The niches were reported in 676 subjects in the single-layer group and 742 women in double-layer group, respectively. Heterogeneity was higher I^2^ = 81%, T^2^ = 0.0479 (Fig. 5). Considering the secondary outcomes, three studies reported the niche depth [28–30], with a range between 2.2 mm ± 1 mm and 6 mm ± 0.1 mm, and the same trial reported the RMT [28–30] (Table 3). Two trials reported the AMT with 8.3–13 mm values for the single layer and 8.1–12.5 mm for the double layer closure [28, 30] (Table 3).

**Table 3:** Secondary outcomes in randomized controlled trials comparing effect of single- vs double-layer uterine closure after three months of Cesarean delivery (CD): depth of niche, residual myometrial thickness (RMT) and adjacent myometrial thickness (AMT)

Outcomes 3 months	Trials (n.refs)	Participants (*n*)	Single-layer closure*	Double-layer closure*
Depth of niche (mm ± DS)	3 [28–30]	2438	2.2 ± 1.0 3.93 (3.81–4.06) 5 ± 0.1	3.7 ± 1.7 3.95 (3.83–4.07) 6 ± 0.1
RMT at US (mm)	3 [28–30]	2438	min–max 5.6–6.4 6.4 7 ± 0.1	min–max 4.2–5.5 6.7 8 ± 0.1
AMT at US (mm) (mm ± DS)	2 [28, 30]	146	8.33 ± 0.7 13 ± 0.1	8.1 ± 1 12.5 ± 0.1

*RMT* residual myometrial thickness, *AMT* adjacent myometrial thickness

#### Six months after cesarean section

**Fig. 6:**
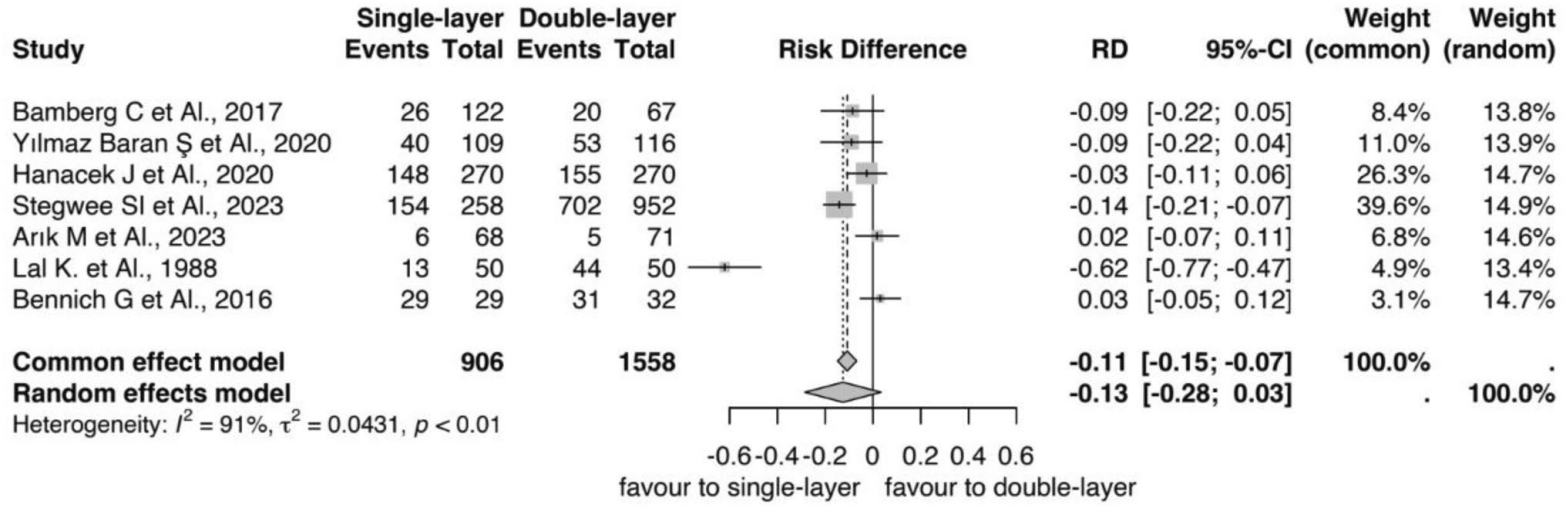
Forest plots showed niche incidence at six months post-cesarean section

Women who received single-layer uterine closure of cesarean delivery incision had a lower incidence of uterine scar defects as did women who received double-layer closure (RD = − 0.11, 95% CI, − 0.15; − 0.07, *p* < 0.01, Fig. 6) in seven trials with 2464 women [23, 24, 26, 27, 31, 32, 34] (906 subjects in single layer group and 1558 in double layer group). The niches were reported in 416/906 subjects in single layer group, and 1010/1558 women in double layer group, respectively (Fig. 6). Heterogeneity was higher *I*^2^ = 91%, *p* < 0.01 (Fig. 4). For secondary outcomes after six months, three RCTs reported the width of niche [24, 31, 35], which ranged from 2.5 to 9 mm. Six trials reported RMT values 6 months after CD, ranging from 3.8 to 9.6 mm for the single layer and 4.3 to 9.4 mm for the double layer [23, 24, 31, 34, 35, 40] (Table 4). Regarding AMT, three trials reported the value, ranging from 8.2 to 12.1 mm for the single layer and 9.4 to 13.15 mm for the double layer [24, 35, 40] (Table 4).

**Table 4:** Secondary outcomes in randomized controlled trials comparing effect of single- vs double-layer uterine closure after six months of Cesarean delivery (CD): depth of niche, residual myometrial thickness (RMT) and adjacent myometrial thickness (AMT)

Outcomes 6 months	Trials (n.refs)	Participants (*n*)	Single-layer closure*	Double-layer closure*
Width of niche (mm ± DS)	3 [24, 31, 35]	490	2.8 (0–7.4) 4.46 ± 1.18 3.4 (2.6–7)	4.0 (0–10.1) 2.63 ± 0.71 9 (2.7–10.4)
RMT at US (mm)	6 [23, 24, 31, 34, 35, 37]	2438	min–max 5.6–6.4 6.4 7 ± 0.1	min–max 4.2–5.5 6.7 8 ± 0.1
Uterine dehiscence in next pregnancy	2 [25, 33]	3325	7/1622	5/1653
AMT at US (mm) (mm ± DS)	2 [28, 30]	146	8.33 ± 0.7 13 ± 0.1	8.1 ± 1 12.5 ± 0.1

*RMT* residual myometrial thickness, *AMT* adjacent myometrial thickness

### Uterine dehiscence in next pregnancy

Three RCTs reported uterine dehiscence in the subsequent pregnancy [36, 37, 39], with eight dehiscences on 1692 pregnant (0.47% of the total) for the single layer and five on 1728 pregnant (0.28% of the total) for the double layer. The main number of patients was recruited by only one study [39].

## Discussion

### Main findings

This systematic review and meta-analysis evaluated the risk of cesarean scar defects after six weeks, three and six months after cesarean delivery. Of the 18 trials included [23–40], 11 were included for the quantitative analysis [23–34]. No difference in cesarean scar defect incidence was found between single-layer closure and double-layer uterine closure after six weeks. After three months, the niche incidence was slightly lower with the single-layer uterine closure technique. After six months, single-layer uterine closure was associated with a significantly lower incidence of cesarean scar defects versus the double-layer group.

### Comparison with existing literature

Previous systematic reviews and meta-analyses assessed single- versus double-layer uterine closure of CD incision and related outcomes. Roberge et al. evidenced no statistical difference in cesarean scar defect and uterine dehiscence [41]. Besides, single-layer uterine closure was associated with significantly lower RMT [41]. According to Qayum et al., single-layer closure evidenced a shorter operative time compared to double-layer closure, while double-layer uterine closure shows more RMT and lower incidence of dysmenorrhea [19]. Di Spiezio Sardo et al. did not reveal any statistically significant difference in the risk of cesarean scar defects in relation to single- versus double-layer closure, with a lower RMT on ultrasound in single-layer closure [18]. They included nine RCTs without a time-based follow-up stratification [18]. Roberge et al. found no significant maternal complications, infection, and blood loss between both techniques in the short term [41]​.

### Strength and limitations

One of the major strengths of this meta-analysis was the RCTs inclusion. Several databases were explored, and more RCTs were included than previous meta-analysis. For the first time, we performed a time-based follow-up stratification, assessing the niche incidence risk after 6 weeks, 3 months, and 6 months. This stratification offers further information about the niche incidence and surgical technique. At the same time, several limitations are present and limit our findings generalizability. Different RCTs included women with multiple cesarean sections, and this condition could impact the niche incidence. A specific subgroup analysis based on significant confounders like parity, suture technique (locking versus nonlocking), inclusion of the decidual layer, and surgical habits was not possible. Similarly, these limitations were evidenced in the previous metanalysis due to a lack of standardized study design [18]. Few trials clearly reported the decidual layer inclusion, type of uterine suture, operative time, intraoperative blood loss, postoperative pain, infection rates, and recovery time. Although these surgical data have biological implications for the myometrial tissue reparation and niches development and represent a physiological inference in the findings analysis. Different RCTs employed locked and unlocked suture techniques for respective single- and double-layer closures, but quantitative analysis was not possible. In addition, the studies reported different approaches to detect the uterine scar. These different imaging methods represent another important gap, and future research should be performed with a standard approach. At least, a limitation is represented by the different follow-ups adopted to assess the scar defect and the different methods adopted to assess the uterine scar defects (by ultrasound or other techniques).

### Clinical and research implications

This metanalysis has significant clinical relevance, especially to minimize the long-term post-cesarean complications. A reduced niche development would minimize the incidence of abnormal uterine bleeding and other complications related to a niche in subsequent pregnancies [42, 43]. It is not entirely evident how these findings are explained biologically. Most likely, the single layer will result in less tissue stress, which could improve cesarean scar healing and reduce the likelihood of uterine scar abnormalities [9, 14, 15]. The single layer technique likely allows for a reduction in the ischemic damage to the endometrial and myometrial tissue. In addition, ischemic damage could cause a defect in healing and recruitment of growth and vascular factors, in favor of an inflammatory infiltrate which could lead to increased tissue damage and fibrosis which determine the onset of a more evident scar and less healing of the uterine tissue [14, 15]. Additional trials with additional data are necessary to further elucidate the long-term risks and benefits of each closure technique, particularly for women desirous of future pregnancies.

## Conclusions

This meta-analysis critically reviewed the niche incidence following cesarean section and evidenced a reduced risk of niche incidence after single-layer versus double-layer uterine closure of cesarean delivery incision after 6 months. The choice of CD closure technique should be tailored to the clinical findings and to the future reproductive wishes of the patient. Finally, additional RCTs will be required to outline long-term outcomes to confirm the reduced uterine scar defect in the single layer and to explore the subsequent pregnancy complications.

## Supplementary Information

Below is the link to the electronic supplementary material.

Supplementary file1 (DOCX 17 KB)

## Data Availability

No datasets were generated or analyzed during the current study.
