# Supplementary material for: Single-versus double-layer uterine closure at the time of cesarean delivery and risk of uterine scar niche: a systematic review and meta-analysis of randomized trials
Source: Arch Gynecol Obstet. 2025 Aug 20;312(4):1095–106. doi: 10.1007/s00404-025-08151-y (PMC12414086; doi:10.1007/s00404-025-08151-y)
Supplement: Supplementary file 1 — Supplementary file1 (DOCX 17 KB) [file 404_2025_8151_MOESM1_ESM.docx]

**APPENDIX 1**

**Detailed search strategy for systematic review.**

| **Variable** | **Search strategy** |
| --- | --- |
| Database searched | PubMed, EMBASE, Scopus, ClinicalTrials.gov and Cochrane Central Register of Controlled Trials from inception of each database until 01 May 2024. |
| Search strategy for Pubmed | (("caesarean"[All Fields] OR "caesareans"[All Fields] OR "cesarean"[All Fields] OR "cesareans"[All Fields]) AND/OR ("deliveries"[All Fields] OR "delivery, obstetric"[MeSH Terms] OR ("delivery"[All Fields] AND "obstetric"[All Fields]) OR "obstetric delivery"[All Fields] OR "delivery"[All Fields])) AND (((("cicatrix"[MeSH Terms] OR "cicatrix"[All Fields] OR "scar"[All Fields]) AND/OR ("abnormalities"[Subheading] OR "abnormalities"[All Fields] OR "defects"[All Fields] OR "defect"[All Fields] OR "defect's"[All Fields] OR "defected"[All Fields] OR "defective"[All Fields] OR "defectively"[All Fields] OR "defectives"[All Fields])) AND/OR ("niche"[All Fields] OR "niche's"[All Fields] OR "niches"[All Fields])) AND/OR ("pouch"[All Fields] OR "pouch's"[All Fields] OR "pouche"[All Fields] OR "pouches"[All Fields] OR "pouching"[All Fields] OR "pouchs"[All Fields])) AND/OR ("dehisce"[All Fields] OR "dehisced"[All Fields] OR "dehiscence"[All Fields] OR "dehiscences"[All Fields] OR "dehiscent"[All Fields] OR "dehisces"[All Fields] OR "dehiscing"[All Fields]) AND ((("uterine closure") AND/OR ("closure"[All Fields] OR "closure's"[All Fields] OR "closures"[All Fields])) OR ("layer"[All Fields] OR "layer's"[All Fields] OR "layered"[All Fields] OR "layering"[All Fields] OR "layerings"[All Fields] OR "layers"[All Fields])) AND (randomizedcontrolledtrial[Filter])[including  pluralization and US English/UK English spelling variations and suffixes/prefixes] |
| EMBASE | (cesarean OR caesarean OR 'obstetric delivery'/exp OR 'obstetric delivery') AND ('scar'/exp OR scar OR defect OR 'niche'/exp OR niche OR pouch OR 'dehiscence'/exp OR dehiscence) AND ('uterine closure'/exp OR 'uterine closure' OR closure OR 'layer'/exp OR layer) |
| Scopus | TITLE-ABS-KEY (cesarean  OR delivery) AND (scar OR defect OR niche OR pouch OR dehiscence) AND (uterine closure OR layer) |
| Cochrane Central Register of Controlled Trials | (cesarean OR delivery) AND (scar OR defect OR niche OR pouch OR dehiscence) AND (uterine closure OR layer) in Title Abstract Keyword - (Word variations have been searched) |
| Clinical Trial | (Cesarean OR delivery) AND (scar OR Scarring OR cicatrix OR defect OR niche OR pouch OR dehiscence) AND (uterine closure OR uterus OR layer) |
| Other sources | The reference lists of selected articles were hand searched to identify any relevant articles. |
